# Association between Food Preferences, Eating Behaviors and Socio-Demographic Factors, Physical Activity among Children and Adolescents: A Cross-Sectional Study

**DOI:** 10.3390/nu12030640

**Published:** 2020-02-28

**Authors:** Chao Qiu, Min Hou

**Affiliations:** 1 College of Humanities, Jiangnan University, 1800 Lihu Road, Wuxi 214122, China; Chao.Qiu@outlook.com; 2 School of Public Health, College of Medicine, Shanghai Jiao Tong University, 227 Chongqing South Road, Shanghai 200025, China

**Keywords:** adolescents, children, eating behavior, food preference, picky eating

## Abstract

The prevalence of overweight and obesity is a serious health issue among children and adolescents worldwide. This study aimed to investigate factors influencing weight status-associated food preferences and eating behaviors. A cross-sectional study was conducted to collect data from 2578 pairs of Chinese children and parents in five cities from December 2018 to March 2019. There was an increase in consumptions of processed seafood, nuts and dried fruit/vegetables, and fruit/vegetable juice, but a reduction of consuming puffed and processed meat products, sugar/artificially sweetened beverages and milk tea, and picky eating. These food preferences differentiate between sexes. Picky eating behavior was greatly presented in children of lower educated mothers or heavy-smoking fathers. Children of the lower educated fathers consumed less processed seafood and dairy products, and those of the heavy-smoking fathers consumed more puffed products, but less fruit/vegetable juice, and had greater snack preference. The father’s body mass index(BMI)status was also positively associated with unhealthy behaviors. Those who exercised daily longer showed better eating behaviors, and picky eating and fast-food lovers likely occurred in higher-income families. Our study provides an insight into that fathers being educated for health-conscious advice and physical activity may be the potential strategies to foster their children’s healthy eating patterns. Their efficacy needs to be further investigated.

## 1. Introduction

The worldwide prevalence of overweight and obesity among children and adolescents has increased remarkably over the past few decades, becoming a global health issue with a growing burden on public healthcare expenditure [1,2]. Data on Chinese children and adolescents shows a tenfold increase in overweight and obesity over the last 30 years, and today, one in five are overweight or obese [3]. Childhood obesity has serious implications for health and diseases, such as hypertension, dyslipidemia, insulin resistance, dysglycemia, fatty liver disease, and psychosocial complications [2], and this is likely to continue in adulthood [4]. For these reasons, it is important to maintain a healthy weight from an early stage. Thus, it is important to identify and regulate which factors influence weight status among children and adolescents in order to control this trend.

Obesity is manifold and complex, being influenced by genetic and non-genetic risk factors [1]. In light of its increasing prevalence among children and adolescents, weight status is generally considered to be a result of unhealthy eating patterns, a lack of physical activity, or a combination of both [2]. Positive associations have been found with the intake of meat [5], fast food [6], and sweetened beverages [7]. Meanwhile, weight status has been inversely associated with the consumption of fruit, vegetables, pulses, and nuts [8]. 

Many studies have demonstrated food preference as one of the critical determinants to food consumption and choice [9,10,11]. There is also growing evidence that food preference is associated with weight status [12,13]. Those with preferences for fatty and sweet foods are more likely to be overweight or obese [11]. In addition to genes, the variety of food preference is substantially shaped by environmental, physiological, and nutritional factors among children and adolescents [14,15,16,17,18,19,20,21,22]. As a result, these factors that determine food preference and eating behaviors subsequently influence specific food consumption, and may then contribute to the development of the overweight and obesity. For children and adolescents, the family setting is the primary environment within which their preferences are developed [23]. However, few studies have been published which investigated the impact of socio-demographic factors on food preference and consumption among Chinese children and adolescents [24]. A clear understanding of the development of food preferences is needed in future studies so that we may use it to promote healthy eating patterns and prevent overweight and obesity.

Picky eating as one of the unhealthy eating behaviors is usually defined as eating a limited variety of foods, and has strongly been linked to food preference [25]. A longitudinal study demonstrated that picky eating emerges at any age [26], and its prevalence varies between 3% and 66% among children and adolescents [27]. Many studies found that picky eating behavior is associated with sex [25,28], household income [29], and maternal age [30]. However, there are inconsistent results with regard to associations with child sex in studies [25,28]. Thus, more studies are needed to investigate factors affecting the development of the picky eating behavior and its consequences on body weight among children and adolescents, especially in China. 

As mentioned above, current data regarding food preference and consumption and unhealthy eating behavior among Chinese children and adolescents is limited. We therefore undertook a cross sectional study to assess impacts of socio-demographic factors, children’s traits, parental characteristics, and breastfed status on food preference and consumption, as well as picky eating behaviors in a large sample of Chinese childhood and adolescents aged 7–16 years old. 

## 2. Materials and Methods 

### 2.1. Study Design and Population

This cross-sectional study recruited children and adolescents from eight primary and secondary schools in five cities including Shanghai, Jiaxing, Dezhou, Suqian, and Fuyang across eastern China from December 2018 to March 2019. The study population was selected based on a proportionate stratified random sampling, taking into account the region with different relative socioeconomic statuses [31] including upper, moderate, and lower levels based on data collected in 2018 by the National Bureau of Statistics of China to provide a regional representative survey of 2nd – 8th grade students from both public and private schools. Sampling took place in classes randomly selected from each grade in the selected schools, where children and their parents were enrolled to complete a series of questionnaires. 

The questionnaires included questions about child age, sex, height, birth weight, delivery mode, mother’s weight gain in pregnancy, breastfeeding history, physical activity, eating behavior, food preference and consumption, parents’ anthropometric measures, education level, smoking status, socio-demographical factors, etc. Those with a serious organ disease, abnormal physical development, or physical impairments were excluded from this study. For the purpose of this study, students were excluded if they were missing data on parent-reported socio-demographic factors, anthropometric and physical information of child and their parents, parental characteristics, breastfeeding, eating behavior, or food preference and consumption. This study was approved by the Ethics Committee of School of Public Health of Shanghai Jiao Tong University for Human Subject Research, and all parents gave written informed consent, No. SJUPN-201815.

### 2.2. Socioeconomic and Anthropometric Measures

Parents reported information about their children, including sex, race/ethnicity, birth date, grade, weight in kilograms, and height in centimeters (without shoes). Parents self-reported family, socio-demographic, pregnancy, and perinatal information as well as breastfeeding information. Parents self-reported their education level, smoking status, household income, and their child’s birth, sex, and breastfeeding history. Parental education was categorized as ‘Secondary high school and lower,’ ‘High school or equivalent,’ and ‘Bachelor degree and higher’. Parental smoking was reported in the questionnaire along with the number of cigarettes smoked per day. Parents were categorized as non-smokers (never), less than five cigarettes per day, 5–10 cigarettes, and more than 10 cigarettes per day. Annual household income was categorized as ‘less than RMB 80,000,′ ‘RMB 80,000-RMB 150,000,′ and ‘more than RMB 150,000′. Regarding the perinatal data, delivery characteristics (full-term, premature, or post-term birth), delivery types (forceps or cesarean section), and birth weight in grams were requested in the questionnaire. Mothers were asked to respond to their age at the time of childbirth, weight gain during pregnancy in kilograms, and the question ‘Did you feed your baby breastmilk?’ Breastfeeding practice was categorized as: ‘not breastfed’, ‘yes, breastfed for <6 months,’ and ‘yes, breastfed for ≥6 months’, if the response was “Yes” at <6 months and “No” at ≥6 months checkup. The 6-month cutoff was according to the World Health Organization breastfeeding guidelines [32].

Participants were encouraged to report weight and height based on annual medical examination or follow instructions of weight and height measurements at home if no annual medical examination, which were subsequently used to calculate body mass index (BMI). Many studies suggest that parent-reported height and weight among children and adolescents, self-reported height and weight, and calculated BMI were in agreement with the measured data [33,34,35]. For parents, BMI was calculated as weight divided by height squared (m^2^). For children and adolescents, the sex- and age-specific BMI cutoff points recommended by the Working Group for Obesity in China (WGOC) [36] were used to define underweight, normal-weight, overweight, and obesity, which were consistent with the Eastern Asia ethnic characteristics of body fatness growth [37] due to the differences in body composition across different ethnic groups. As there were too few obese children (1.1%), we combined obese and overweight (12.7%) into one category (overweight/obesity) in all analyses. 

### 2.3. Physical Activity

Questions on physical activity for children were modified from the NHANES study [38]. The questionnaire was designed to collect information on moderate physical activities (walking) and vigorous physical activities (running, basketball/soccer, swimming, cycling, skating, dancing, self-defense, and jogging) during a typical week. Time spent on physical activities mentioned above was assessed by the question, “Average hours per day your child spent on physical activities over the past 30 days?” Response options (hours) were less than 1, 1, 2, 3, or 4, 5 or more, and none. Less than 1 h was coded as 0.5 h and 5 h or more was coded as 5 h. Those who took less than 30 min daily exercise were considered as not physically active. 

### 2.4. Eating Habits Questionnaire

#### 2.4.1. Food Preference

Parents completed a food preference question by asking their children composing of a list of written descriptions of three classes of common food and beverage items, such as meals including meat (e.g., pork, beef, and lamb), vegetables, fast food (e.g., hamburgers and fried chicken), and snacks instead of main meals (e.g., cakes, biscuits, candy, chocolate, jelly, puffed products, processed meat/seafood products, dairy products). Parents were asked to make an assessment as to whether or not they would like to eat it by responding “yes” or “no.” They were instructed to subjectively consider food items, and to limit their thinking time for any one of the food items. The responses for each food item were scored 0 or 1 to describe preference or non-preference, respectively.

#### 2.4.2. Picky Eating Behavior 

A single question on picky eating for their children was modified from the ALSPAC study [39]. The question was “she/he was choosy about certain food (like meat, vegetables, fruits etc.) she/he ate”. Response options were ‘seldom or never’ or ‘yes, very choosy’ [40]. The responses for each food item were scored 0 or 1 to describe the children as not picky or picky.

#### 2.4.3. Food Frequency

Food consumption habits for their children were evaluated by asking questions about healthy/unhealthy food consumption choices, including fast food, snacks consisting of sweets (e.g., cakes, biscuits, candy, chocolate, jelly and preserved fruits), puffed products (e.g., chips), processed meat products (e.g., ham and sliced dried beef), processed seafood products (e.g., sliced fish), dairy products (e.g., cream, cheese, ice-cream), nuts/dried fruits/vegetables (e.g., nuts, dried fruits, and vegetables), and beverages including sugar-sweetened beverages (e.g., carbonated drinks and tea drinks), dairy beverages, artificially sugar-sweetened beverages, sugar-sweetened milk tea, tea, and water. The consumption frequency was evaluated on a three-point scale, and subsequently categorized as less than once a week, 2–3 times a week, and more than 4 times a week. Among these point scales, more than 4 times a week was considered high consumption.

### 2.5. Statistical Analysis

We obtained the means and standard deviations (SD) for continuous variables: age, BMI and physical activity time, and frequencies and percentages for the categorical variables: breastfeeding duration, education level, smoking, and household income, and within strata defined by gender. To examine differences of continuous variables between boys and girls, *t*-tests were used, whereas the chi-square test was used to compare differences of categorical variables. 

In binary logistic regression models, we estimated crude odds ratios (ORs) with 95% confidence intervals (95% CI) for the associations of food preference and consumption, and picky eating behavior (e.g., preferences for meat, vegetables, snacks and fast food, picking eating behavior, consumption of foods including sweets, puff products, processed meat products, processed seafood, dairy products, nuts/dried fruits/vegetables, sugar-sweetened beverage, dairy beverage, artificially sugar-sweetened beverage, sugar-sweetened milk tea, tea, and water) with socio-demographic, perinatal variables (age, gender, breastfeeding history, parental BMI, education level and smoking status, household income) and child’s physical activity duration, using the category of “girls,” “breastfeeding more than 6 months,” “bachelor and higher education,” “never smoke,” and “less than RMB 80000” as a reference. 

In multiple linear regression models, we estimated β-coefficient with 95% CI for the associations of consumption frequency of fast food, snacks, and beverages with age, gender, breastfeeding history, parental BMI, parental education, parental smoking, household income, and child’s physical activity duration. Tests for linear trend by entering each category as a continuous variable in the linear regression models. Analyses were completed with the IBM SPSS program, version 22 (IBM, Chicago, IL, USA). Two-sided *p* < 0.05 was considered to be statistically significant.

## 3. Results

### 3.1. Participants

In total, data was collected from 2578 children and adolescents (1303 boys and 1275 girls) aged 7–16 years (Mean age  =  12.8  ±  2.2) (70% response rate) in Table 1, in which the child’s age, race, weight, height, BMI, physical activity, breastfeeding, parental BMI, education levels, smoking status, and household income are shown. About half (50.5%) of children and adolescents were boys in this study. The majority was ethnically Han Chinese (98.8%), and had a normal weight (70.5%). Using WGOC criteria, the prevalence of underweight, overweight, and obesity were 15.7%, 12.7%, and 1.1%, respectively. We found a higher prevalence of overweight and obesity among boys than girls (*p* < 0.0001). The majority of mothers had a normal weight (72%), while most of the fathers were overweight or obese (54.5%). In total, 31.7% of the mothers and 34.5% of the fathers obtained a bachelor degree or a higher educational level, and 99.3% of the mothers and half of the fathers were non-smokers. Household income was not significantly different between families of boys and girls (*p* = 0.28). Longer daily exercise was reported amongst the boys, but less than 1 h per day (*p* < 0.05). 

### 3.2. Associations between Preferences for Meat, Vegetables, Snacks, Fast Food, and Factors

The results examining factors impacting preferences for meat and vegetables are presented in Table 2. Boys more likely preferred meat than girls (*p* < 0.001). Additionally, boys and girls of a father who smoked more than 10 cigarettes per day had less preference for vegetables compared those with a father who was non-smoker (*p* = 0.04). 

Among variables in Table 3, age was negatively associated with snacking preference (*p* < 0.0001). Children of mothers who obtained the lowest education level had a higher risk of snack preference than those of mothers who had attained bachelor’s degrees or a higher level of education (*p* = 0.05). Dose-response analysis showed that children and adolescents of fathers who daily smoked more cigarettes tended to prefer snacks (*p* for trends < 0.001) and fast food (*p* for trends = 0.05). Furthermore, those who were breastfed for less than 6 months were more likely to prefer snacks than those who were breastfed for more than 6 months (*p* = 0.04). Physical activity had a slight inverse correlation with preferences for meat, snacks, and fast food (all *p*-values < 0.04).

### 3.3. Associations between Picky Eating Behavior and Factors

Table 4 shows the associations between picky eating and the child’s age, gender, parental BMI, education levels, smoking status, breastfeeding duration, household income, and physical activity duration. Younger boys were more likely to be picky eaters (*p* < 0.001). Dose-response analysis showed that children and adolescents whose mother received a lower education, whose father smoked more cigarettes per day, or who were from a higher income family were more likely to develop picky eating behavior (*p* value for all trends < 0.05). The average physical activity duration had a weak negative association with picky eating behaviors amongst the students who participated in the study. 

### 3.4. Associations between Consumptions of Various Snacks and Factors

Consumption of puffed products and processed meat products were observed to be more likely among elder subjects, while younger ones chose to eat processed seafood and nuts/dried fruits/vegetables (all *p* < 0.01) in Table 5. Boys consumed less sweets and processed seafood (adjusted Odds Ratio, 0.53 (0.41–0.70); 0.61 (0.47–0.78)), but more processed meat products (adjusted Odds Ratio, 1.28 (1.03–1.61) than girls. Children and adolescents of mothers who had completed secondary school had more puffed products, while those of mothers with lower than a secondary school education tended to consume less dairy products in comparison with the children of mothers who had a bachelor’s degree or higher level of education. Associations between the father’s education level and intakes of processed seafood and dairy products were found, and presented a decreasing trend: children of higher educated fathers consumed less processed seafood and dairy products (all *p* for trends < 0.03). The prevalence of consuming puffed products increased in fathers who smoked more cigarettes (*p* for trends < 0.02). Children and adolescents consumed nuts/dried fruits/vegetables less than other snacks when their fathers smoked more than 10 cigarettes per day compared with those whose fathers were non-smokers (adjusted Odds Ratio, 0.70 (0.49–0.99)). Furthermore, there was a slight negative association between physical activity and consumption of puffed products. Children who daily performed longer physical activity tended to consume less puffed products, but more nuts/dried fruits/vegetables amongst all ages.

### 3.5. Associations between Consumptions of Various Beverages and Factors

Table 6 shows increased consumption of sugar-sweetened beverages, artificially sweetened beverages and sugar sweetened milk teas as children aged, but decreased preference for fruit/vegetable juice (all *p* < 0.01). Boys were more likely to drink sugar-sweetened beverages, but less sugar sweetened milk tea compared to girls (all *p* < 0.001). Apart from age and gender, selection of beverages seems to be slightly influenced by other factors. However, children and adolescents of fathers who smoked more cigarettes tended not to consume fruit/vegetable juice (*p* for trends <0.001) and fast food (*p* for trends = 0.02). In addition, there was a decreasing consumption of tea in students from higher income families (*p* for trends = 0.02). A slight positive association was found between physical activity duration and consumption of artificially sweetened beverages among children and adolescents.

### 3.6. Associations between the Consumption Frequencies of Fast Food, Snacks, Beverages, and Factors

The frequency of the intake of fast food, snacks, and beverages increased with age among children and adolescents (*p* for all trends < 0.001) (Table 7). Girls had snacks more often than boys (*p* for trends = 0.01). The mother’s BMI status was inversely associated with times of their children’s exposure to snacks (*p* for trends = 0.02), while a positive association between frequency of fast food intake among children and adolescents and increasing father’s BMI was observed (*p* for trends = 0.02). Children and adolescents of mothers with higher education levels consumed beverages at a lower frequency (*p* for trends < 0.001). Children and adolescents of fathers who smoked more cigarettes and from higher income families consumed snacks more often (*p* for trends < 0.001). There was a slight positive association between longer daily physical activity duration and times of consumption of snacks and beverages (*p* for trends < 0.001).

## 4. Discussion

In this cross-sectional study, we observed factors influencing food preferences, picky eating behaviors, and food consumption frequency among children and adolescents in eastern China. We found a higher prevalence of overweight and obesity among boys than girls. The daily physical activity was found to be slightly longer in boys than girls. Those with a longer daily exercise had less consumption frequency of snacks and fast food and were less likely to be picky eaters. Preference for meat was associated with gender: males were more likely to prefer meat. Girls consumed more sweets, processed seafood, and nuts/dried fruits/vegetables, and displayed a preference for sugar sweetened milk tea compared to sugar-sweetened beverages. The younger had less preferences for puffed products, processed meat products, processed seafood and nuts/dried fruits/vegetables when they consumed snacks, and was inversely associated with consumption frequency of sugar-sweetened beverages, artificially sweetened beverages, and sugar sweetened milk teas, but was positively linked to fruit/vegetable juice. Children with picky eating behavior were more often found in families with less educated mothers, or fathers who smoked more cigarettes. In addition, the more the fathers smoked, the more likely their child preferred snacks, fast food, and puffed products, but less fruit/vegetable juice. Children whose fathers were less educated tended to eat less processed seafood and dairy foods. In addition, more frequent consumption of fast food, snacks, and beverages was associated with fathers with higher BMI and heavier smoking status and being from families with higher income, as well as having longer daily physical activity. Furthermore, increasing household income had a positive relationship with likelihood of picky eating behavior, preference for fast food and the intake of tea, but less frequently consuming sugar-sweetened beverages. Breastfeeding likely attenuated the preference for snacks. Children and adolescents who were breastfed for a shorter duration and those who were more physically active tended to prefer nuts/dried fruits/vegetables and drink artificially sweetened beverages. Our results add to the evidence that maternal feeding practices [41], food choice motives [42] and parents’ characteristics, especially fathers’ smoking status, play a crucial role in shaping food preference and consumption habit as well as healthy eating behaviors among children and adolescents in China. 

Our findings, akin to those of previous studies [43,44], found that there were age-related differences in food preferences and picky eating behaviors. Among the items we studied, the intake of puffed products, processed meat products, sugar sweetened beverages, artificially sweetened beverages, and sugar-sweetened milk tea were more likely to occur in younger children. Inversely, older children consumed more processed seafood products, nuts and dried fruit/vegetables, and fruit/vegetable juice. Furthermore, our study suggests that research on picky eating behaviors should focus on younger children. This would have an implication that the conception and behavior of healthy eating increases among children and adolescents while they are growing. Thus, more studies are needed to provide dietary guidance on younger. 

In our study, boys tended to consume processed meat products. Consistent with previous findings, boys had a greater preference for fatty foods than girls [45,46,47]. This may be attributed to boys’ greater energy requirements by eating more energy-dense foods in comparison with girls [47]. Meanwhile, we have made the first observation that girls consumed more processed seafood than boys. As scientific research provides insights into the functioning of seafood [48], there is a growing awareness that processed seafood may be healthier and contains very little energy compared to other processed foods to affect the overall health of the consumer. This may be a reason for the finding above. Additionally, we found that the girls showed a greater preference for sweets than boys, which is supported by a previous study [46]. One possible explanation from previous research suggested that the sweets rich in simple sugars are regarded as comfort foods by girls [49]. Our results add to the evidence that boys consumed more sugar-sweetened beverages, while girls were more likely to have sugar sweetened milk tea. Even though drinking sugar sweetened milk tea may cause negative effects on health due to a large amount of sugar, it is a very popular drinking lifestyle of Chinese girls at present. Thus, its negative impact on girls’ health should be studied in future research. Furthermore, boys in this study were more likely to be picky eaters, as suggested by Cooke [47]. Thus, strategies for the prevention or intervention to combat negative eating behaviors should tailor messages appropriately to target specific subjects by age and gender.

In our study, the children of mothers with a lower level of education, who tend to be much less health-literate [50], were more likely to become picky eaters. Interestingly, we found that the children of less-educated fathers ate less processed seafood and dairy foods that are good sources of protein and calcium, with adequate energy and fat content [51]. Children of fathers who smoked more cigarettes were more likely to become picky eaters, and displayed a stronger preference for snacks, tended to eat more puffed products, and drank less fruit/vegetable juice as well as consuming fast food, snacks, and sugary beverages at a greater frequency. These behaviors were also associated with increasing BMI of the father. Evidence suggesting an association between parents’ general interest in health and healthier food choice behaviors [52] provides an important insight into the impact of father’s healthy behaviors on children’s eating behavior. Previous studies have examined how parents may impact on children’s food preferences [53,54] and eating behaviors [55,56,57]. A meta-analysis of 37 studies suggested that parental restrictive guidance/rule-making was effective at preventing unhealthy eating in children aged 7 or older [58]. In a study of 108 preschool-aged children and their parents, Vollmer et al. found that preference for vegetables mostly occurred among children of parents with the authoritative feeding style (high demands, responds to child’s needs), but its association with healthy eating was moderated by food parenting practices [54]. In addition, a longitudinal study of approximately 5000 children found that in early childhood, children with authoritative and permissive fathers, or girls with authoritative mothers, were more likely to consume fruits and vegetables in late childhood [59]. Although we did not assess food practices and feeding styles of the fathers, it can be implied that fathers play an important role in development of their children’s eating behaviors. Therefore, we suggest that much more work should be done to target parents when developing and implementing interventions to foster children’s healthy eating patterns and tackle related health conditions. It may be especially important to target fathers, who are traditionally seen as the educator and disciplinarian in the Chinese family.

Our study found that children from high-income households tended to prefer fast food and were less likely to drink tea in addition to more frequently being picky eaters. This is in line with the popularity of fast food among children and adolescents with the growth of family income in China. The regular consumption of these foods may lead to an excess of daily energy intake, leading to the greater prevalence of overweight and obesity among children and adolescents in China today. Thus, some action should be taken to prevent frequent exposure to fast foods such as burgers and chips that are typically high in saturated fat and low in micronutrients [60]. Considering less consumption of vegetable and fruits among children and adolescents, dietary nutritional education and intervention about healthy eating is necessary. 

As seen in this study, daily average physical activity duration was slightly associated with preferences and consumption for certain foods as well as picky eating behaviors among children and adolescents. In a study based on three cross-sectional surveys during the years of 2009, 2012, and 2015 in Brazil, a positive tendency to practice physical activities with healthy eating was shown among children between 7 and 14 years [61]. Thus, our results may support the effects of physical activity on eating behaviors, although the extent to play a role needs to be investigated further.

To the best of our knowledge, this study was the first to assess variable factors in a large sample of parents and their children in China, which allowed for multivariate analyses of correlates of child’s food preference and eating behaviors. However, the study has limitations. Firstly, the parents were self-reporting the data, which increases the likelihood that results are affected by reporting bias. Secondly, there is a need to investigate the impacts longitudinally across all age spans in childhood across China. Furthermore, it may be more informative to have details about food parenting practices and feeding styles as well as caregivers. 

## 5. Conclusions

Because of the overweight and obesity problem among children and adolescents, much attention has been given to their eating patterns. Our results demonstrate that characters of parents, household income and physical activity are associated with children’s eating behaviors independently of age and sex. To prevent and ameliorate unhealthy eating behaviors among children and adolescents, effective strategies should therefore precisely target to individuals through the implementation of interventions such as nutrition and health education as well as physical activities. The approaches and their efficacy in preventing unhealthy eating behaviors must be evaluated in future research, to improve children’s eating behaviors and reduce the potential burden of the eating behavior-related illnesses, such as obesity on China’s healthcare system.

## Figures and Tables

**Table 1:** Participants’ Characteristics.

Characteristic	Total (*n* = 2578)		Boys (*n* = 1303)		Girls (*n* = 1275)		*p*
	Mean (SD)	n (%)	Mean (SD)	n (%)	Mean (SD)	n (%)	
**Age**	12.7(2.2)	-	12.7 (2.3)	-	12.9 (2.1)	-	**0.01**
**Race**							
**Han**	- -	2505 (98.8)	- -	1254 (50.1)	- -	1251 (49.9)	0.28
**Minority**		31 (1.2)		19 (61.3)		12 (49.8)	
**Weight (kg)**	45.3 (11.8)	-	46.7 (12.8)	-	43.8(10.5)	-	**0.00**
**Height (cm)**	155.1 (13.6)	-	156.3 (15.1)	-	153.8(11.9)	-	**0.00**
**Children BMI category ^a^**							
**Underweight**	-	405 (15.7)	-	175 (43.2)	-	230 (56.8)	**0.00**
**Normal**	-	1817 (70.5)	-	881 (48.5)	-	936 (51.5)	
**Overweight**	-	328 (12.7)	-	226 (68.9)	-	102 (31.1)	
**Obese**	-	28 (1.1)	-	21 (75)	-	7 (25)	
**Breastfeeding (months)**							
**0**	-	370 (16)	-	185 (50)	-	185 (50)	0.80
**<6**	-	364 (15.7)	-	183 (50.3)	-	181 (49.7)	
**≥6**	-	1578 (68.3)	-	800 (50.7)	-	778 (49.3)	
**Mother BMI**	22.2 (2.7)	-	22.2 (2.6)	-	22.2 (2.8)	-	0.74
**Father BMI**	24.5 (3.3)	-	24.6 (3.3)	-	24.4 (3.3)	-	0.15
**Mother Smoking (cigarettes/day)**							
**0**	-	2208 (99.3)	-	1103 (50.0)	-	1105 (50.0)	1.00
**≥1**	-	15 (0.7)	-	6 (40.0)	[	9 (60.0)	
**Father Smoking (cigarettes/day)**							
**0**	-	1103 (49.6)	-	555 (50.3)	-	548 (49.7)	0.58
**<5**	-	592 (26.6)	-	278 (47)	-	314 (53)	
**5–10**	-	308 (13.9)	-	164 (53.2)	-	144 (46.8)	
**>10**	-	219 (9.9)	-	117 (53.4)	-	102 (46.6)	
**Mother Education, n (%)**							
**Secondary high school or lower**	-	945 (36.7)	-	507 (53.7)	-	438 (46.3)	0.06
**High school**	-	657 (25.5)	-	318 (48.4)	-	339 (51.6)	
**Bachelor’s or higher**	-	818 (31.7)	-	404 (49.4)	-	414 (50.6)	
**Father Education, n (%)**							
**Secondary high school or lower**	-	717 (27.8)	-	370 (51.6)	-	347 (48.4)	0.68
**High school**	-	812 (31.5)	-	405 (49.9)	-	407 (50.1)	
**Bachelor’s or higher**	-	890 (34.5)	-	449 (50.4)	-	441 (49.6)	
**Household Income (RMB)**							
**<80,000**	-	594 (25.7)	-	288 (48.5)	-	306 (51.5)	0.28
**80,000–150,000**	-	924 (39.9)	-	456 (49.4)	-	468 (50.6)	
**>150,000**	-	795 (34.4)	-	408 (51.3)	-	387 (38.7)	
**Physical Activity (min/day)**	22.9 (27.9)	-	24.0 (29.7)	-	21.7 (25.9)	-	**0.04**

n (%) reported for dichotomous variables; Mean (SD) reported for continuous variables; Boldface indicates statistical significance (*p* < 0.05); ^a^: Cut off classifications for children and adolescents based on the Working Group for Obesity in China (WGOC) [36]; SD: standard deviation; BMI: body mass index.

**Table 2:** Associations between preferences for meat and vegetables and factors from logistic regression model.

Variables	Meat ^a^				Vegetables ^a^			
	OR	95% CI	*p*-Value	*p*-Trend	OR	95% CI	*p*-Value	*p*-Trend
**Age**	1.02	0.96–1.08	0.56		1.05	0.93–1.18	0.44	
**Sex**								
**Girls (ref.)**	-	-	-		-	-	-	
**Boys**	0.60	0.47–0.78	0.00		0.88	0.52–1.48	0.62	
**Mother BMI**	1.02	0.97–1.07	0.50		1.00	0.91–1.11	0.93	
**Father BMI**	1.01	0.97–1.05	0.54		1.03	0.95–1.12	0.46	
**Mother Education**								
**Secondary high school or lower**	0.71	0.47–1.07	0.10	0.26	1.03	0.44–2.41	0.94	1.00
**High school**	0.86	0.59–1.25	0.42		1.02	0.46–2.26	0.54	
**Bachelor’s or** **higher (ref.)**	-	-	-		-	-	-	
**Father Education**								
**Secondary high school or lower**	1.38	0.90–2.11	0.14	0.32	0.57	0.24–1.37	0.21	0.45
**High school**	1.24	0.87–1.77	0.24		0.76	0.35–1.65	0.48	
**Bachelor’s or** **higher (ref.)**	-	-	-		-	-	-	
**Mother Smoking (cigarettes/day)**								
**0**	-	-	-		-	-	-	
**≥1**	2.09	0.26–16.97	0.49		NA	NA	1.00	
**Father Smoking (cigarettes/day)**								
**0 (ref.)**	-	-	-	0.60	-	-	-	0.14
**<5**	0.82	0.61–1.11	0.21		1.02	0.53–1.95	0.97	
**5–10**	0.85	0.59–1.25	0.42		1.12	0.48–2.64	0.79	
**>10**	0.96	0.62–1.49	0.86		0.47	0.23–0.95	0.04	
**Household income (RMB)**								
**<80,000 (ref.)**	-	-	-	0.27	-	-	-	0.74
**80,000–150,000**	0.84	0.59–1.19	0.32		0.85	0.42–1.72	0.65	
**>150,000**	0.73	0.50–1.07	0.11		0.74	0.35–1.58	0.44	
**Breastfeeding (months)**								
**0**	0.89	0.63–1.24	0.48	0.62	0.90	0.45–1.78	0.76	0.89
**< 6**	1.11	0.77–1.59	0.59		1.14	0.52–2.49	0.89	
**≥6 (ref.)**	-	-	-		-	-	-	
**Physical activity (minutes/day)**	1.01	1.00–1.01	0.01		1.00	0.99–1.01	0.65	

^a^ non-preference as reference; NA, not applicable; OR: Odds Ratio; CI: Confidence intervals.

**Table 3:** Associations between preferences for snacks and fast food and factors from logistic regression model.

Variables	Snacks ^a^				Fast Food ^a^			
	OR	95% CI	*p*-Value	*p*-Trend	OR	95% CI	*p*-Value	*p*-Trend
**Age**	1.24	1.15–1.34	0.00		1.03	0.98–1.08	0.24	
**Sex**								
**Girls (ref.)**	-	-	-		-	-	-	
**Boys**	0.94	0.66–1.35	0.73		0.86	0.69–1.06	0.15	
**Mother BMI**	0.99	0.92–1.05	0.67		0.98	0.94–1.02	0.33	
**Father BMI**	1.02	0.96–1.08	0.58		0.99	0.96–1.03	0.77	
**Mother Education**								
**Secondary high** **school or lower**	0.55	0.31–1.00	0.05	0.13	1.26	0.90–1.78	0.18	0.24
**High school**	0.77	0.44–1.33	0.35		1.29	0.95–1.77	0.11	
**Bachelor’s or** **higher (ref.)**	-	-	-		-	-	-	
**Father Education**								
**Secondary high school or lower**	0.99	0.55–1.80	0.98	0.98	1.07	0.75–1.53	0.69	0.83
**High school**	1.04	0.62–1.75	0.88		1.10	0.81–1.48	0.55	
**Bachelor’s or** **higher (ref.)**	-	-	-		-	-	-	
**Mother Smoking (cigarettes/day)**								
**0 (ref.)**	-	-	-		-	-	-	
**≥1**	1.31	0.16–11.07	0.80		1.87	0.55–6.32	0.32	
**Father Smoking (cigarettes/day)**								
**0 (ref.)**				0.00				0.05
**<5**	1.68	1.03–2.75	0.04		0.88	0.69–1.13	0.33	
**5–10**	1.07	0.61–1.86	0.82		0.69	0.49–0.96	0.03	
**>10**	0.49	0.30–0.81	0.01		0.68	0.47–0.98	0.04	
**Household income (RMB)**								
**<80,000 (ref.)**	-	-	-	0.47	-	-	-	0.05
**80,000–150,000**	0.79	0.48–1.28	0.34		0.73	0.55–0.96	0.03	
**>150,000**	0.72	0.43–1.22	0.23		0.72	0.53–0.98	0.04	
**Breastfeeding (months)**								
**0**	0.76	0.48–1.22	0.26	0.04	0.92	0.68–1.23	0.56	0.84
**< 6**	0.55	0.34–0.89	0.02		0.98	0.72–1.32	0.88	
**≥ 6 (ref.)**	-	-	-		-	-	-	
**Physical activity (minutes/day)**	1.01	1.00–1.02	0.04		1.01	1.00–1.01	0.01	

^a^ non-preference as reference.

**Table 4:** Associations between being picky eating and factors from logistic regression model.

Variables	OR	95% CI	*p*-Value	*p*-Trend
**Age**	0.89	0.85–0.94	0.00	
**Sex**				
**Girls (ref.)**	-	-	-	
**Boys**	1.52	1.23–1.90	0.00	
**Mother BMI**	0.99	0.95–1.04	0.73	
**Father BMI**	0.98	0.95–1.01	0.26	
**Mother Education**				
**Secondary high school or lower**	1.61	1.13–2.30	0.01	0.03
**High school**	1.24	0.89–1.71	0.20	
**Bachelor’s or higher (ref.)**	-	-	-	
**Father education**				
**Secondary high school or lower**	0.87	0.60–1.26	0.47	0.67
**High school**	0.87	0.64–1.20	0.40	
**Bachelor’s or higher (ref.)**	-	-	-	
**Mother smoking (cigarettes/day)**				
**0 (ref.)**	-	-	-	
**≥1**	0.42	0.09–2.03	0.28	
**Father smoking (cigarettes/day)**				
**0 (ref.)**				0.02
**<5**	0.98	0.75–1.28	0.89	
**5–10**	1.08	0.77–1.50	0.67	
**>10**	1.73	1.20–2.47	0.00	
**Household income (RMB)**				
**< 80,000 (ref.)**	-	-	-	0.05
**80,000–150,000**	1.28	0.95–1.72	0.11	
**> 150,000**	1.50	1.08–2.07	0.02	
**Breastfeeding (months)**				
**0**	1.23	0.91–1.66	0.17	0.33
**< 6**	1.14	0.83–1.55	0.41	
**≥ 6 (ref.)**	-	-	-	
**Physical activity (minutes/day)**	0.99	0.98–0.99	0.00	

Picky eating as reference.

**Table 5:** Results from logistic regression analyses showing relationships between consumptions of various snacks and factors.

Variables	Sweets ^a^				Puff Products ^a^				Processed Meat Products ^a^				Processed Seafood ^a^				Dairy Products ^a^				Nuts & Dried Fruit/Vegetable ^a^			
	OR	95% CI	*p*-Value	*p*-Trend	OR	95% CI	*p*-Value	*p*-Trend	OR	95% CI	*p*-Value	*p*-Trend	OR	95% CI	*p*-Value	*p*-Trend	OR	95% CI	*p*-Value	*p*-Trend	OR	95% CI	*p*-Value	*p*-Trend
**Age**	1.03	0.97–1.09	0.39		1.07	1.02–1.13	0.01		1.09	1.03–1.15	<0.01		0.88	0.83–0.93	<0.001		1.03	0.98–1.08	0.29		0.91	0.86–0.95	<0.001	
**Sex**																								
**Girls (ref.)**	-	-	-		-	-	-		-	-	-		-	-	-		-	-	-		-	-	-	
**Boys**	0.53	0.41–0.70	0.00		0.91	0.74–1.11	0.35		1.28	1.03–1.61	0.03		0.61	0.47–0.78	<0.001		0.94	0.77–1.15	0.55		0.76	0.61–0.93	0.01	
**Mother BMI**	0.98	0.93–1.03	0.41		1.02	0.98–1.06	0.44		1.01	0.96–1.05	0.78		0.98	0.94–1.03	0.50		1.01	0.97–1.05	0.71		0.98	0.95–1.02	0.44	
**Father BMI**	1.01	0.97–1.05	0.76		1.02	0.99–1.05	0.30		1.00	0.97–1.04	0.95		1.00	0.96–1.04	0.86		0.97	0.94–1.00	0.06		0.99	0.96–1.02	0.38	
**Mother Education**																								
**Secondary high school or lower**	1.22	0.79–1.88	0.37	0.67	1.11	0.79–1.55	0.54	0.10	1.03	0.72–1.47	0.89	0.91	1.28	0.86–1.90	0.23	0.06	**0.67**	**0.49–0.93**	**0.02**	0.06	0.83	0.59–1.17	0.29	0.52
**High school**	1.12	0.75–1.66	0.59		1.37	1.01–1.85	0.04		0.96	0.69–1.33	0.80		0.82	0.57–1.19	0.30		0.83	0.62–1.12	0.22		0.96	0.71–1.31	0.80	
**Bachelor’s or higher (ref.)**	-	-	-		-	-	-		-	-	-		-	-	-		-	-	-		-	-	-	
**Father Education**																								
**Secondary high school or lower**	1.01	0.64–1.60	0.96	0.98	0.92	0.65–1.31	0.65	0.84	0.95	0.65–1.38	0.78	0.58	0.54	0.35–0.83	<0.01	<0.01	0.66	0.47–0.92	0.02	0.03	1.17	0.82–1.66	0.39	0.61
**High school**	1.04	0.71–1.53	0.84		0.92	0.69–1.23	0.57		1.11	0.81–1.52	0.52		0.90	0.64–1.28	0.56		0.92	0.69–1.22	0.57		1.02	0.76–1.37	0.91	
**Bachelor’s or higher (ref.)**	-	-	-		-	-	-		-	-	-		-	-	-		-	-	-		-	-	-	
**Mother Smoking (cigarettes/day)**																								
**0 (ref.)**	-	-	-		-	-	-		-	-	-		-	-	-		-	-	-		-	-	-	
**≥1**	0.77	0.16–3.73	0.75		1.50	0.44–5.05	0.51		1.09	0.28–4.27	0.90		3.05	0.88–10.52	0.08		0.52	0.15–1.82	0.30		0.56	0.16–1.90	0.35	
**Father Smoking (cigarettes/day)**																								
**0 (ref.)**	-	-	-	0.77	-	-	-	**0.02**	-	-	-	0.69	-	-	-	0.22	-	-	-	0.19	-	-	-	0.20
**<5**	0.94	0.68–1.29	0.69		1.26	0.98–1.62	0.07		1.04	0.80–1.36	0.75		1.37	1.02–1.85	0.04		1.02	0.80–1.30	0.90		1.10	0.86–1.43	0.45	
**5–10**	1.11	0.73–1.68	0.62		1.21	0.88–1.65	0.24		0.90	0.64–1.27	0.55		1.21	0.82–1.78	0.34		1.40	1.03–1.91	0.03		1.05	0.76–1.44	0.77	
**>10**	1.19	0.74–1.91	0.48		1.67	1.18–2.37	<0.01		0.83	0.56–1.24	0.37		1.18	0.76–1.84	0.47		1.09	0.77–1.54	0.64		0.70	0.49–0.99	<0.05	
**Household income (RMB)**																								
**< 80,000 (ref.)**	-	-	-	0.18	-	-	-	0.08	-	-	-	0.39	-	-	-	0.97	-	-	-	0.08	-	-	-	0.55
**80,000–150,000**	1.37	0.96–1.95	0.08		**1.38**	**1.04–1.83**	**0.03**		1.22	0.90–1.66	0.20		1.04	0.73–1.47	0.82		1.29	0.98–1.70	0.07		0.97	0.73–1.29	0.84	
**> 150,000**	1.36	0.93–2.01	0.12		1.20	0.88–1.63	0.25		1.23	0.88–1.722	0.22		1.04	0.71–1.52	0.83		1.40	1.04–1.89	0.03		1.12	0.82–1.52	0.49	
**Breastfeeding (months)**																								
**0**	1.16	0.80–1.70	0.44	0.62	1.23	0.92–1.63	0.16	0.20	1.07	0.79–1.46	0.64	0.11	1.26	0.91–1.76	0.17	0.16	1.11	0.84–1.47	0.47	0.72	0.69	0.51–0.91	0.01	<0.01
**<6**	0.91	0.64–1.33	0.63		1.22	0.92–1.63	0.17		0.73	0.52–1.01	0.06		0.82	0.57–1.20	0.31		1.07	0.81–1.43	0.62		0.66	0.50–0.88	0.01	
**≥6 (ref.)**	-	-	-		-	-	-		-	-	-		-	-	-		-	-	-		-	-	-	
**Physical activity (minutes/day)**	1.00	0.99–1.00	0.32		0.99	0.99–1.00	<0.01		1.00	1.00–1.00	0.96		1.00	1.00–1.01	0.83		1.00	0.99–1.00	0.86		1.01	1.00–1.01	<0.01	

^a^ consumption as reference.

**Table 6:** Results from logistic regression analyses showing associations between consumptions of various beverages and factors.

Variables	Sugar Sweetened Beverage ^a^				Artificially Sweetened Beverage ^a^				Sugar Sweetened Milk Tea ^a^				Dairy Beverage ^a^				Tea ^a^				Fruit/Vegetable Juice ^a^			
	OR	95% CI	*p*-Value	*p*-Trend	OR	95% CI	*p*-Value	*p*-Trend	OR	95% CI	*p*-Value	*p*-Trend	OR	95% CI	*p*-Value	*p*-Trend	OR	95% CI	*p*-Value	*p*-Trend	OR	95% CI	*p*-Value	*p*-Trend
**Age**	1.23	1.16–1.30	<0.001		1.18	1.03–1.35	0.01		1.19	1.12–1.26	<0.001		0.95	0.91–1.00	0.06		0.98	0.90–1.06	0.54		0.88	0.83–0.93	<0.001	
**Sex**																								
**Girls (ref.)**	-	-	-		-	-	-		-	-	-		-	-	-		-	-	-		-	-	-	
**Boys**	1.82	1.46–2.25	<0.001		0.73	0.46–1.16	0.18		0.61	0.49–0.76	<0.001		1.06	0.87–1.30	0.58		0.87	0.61–1.24	0.45		0.86	0.66–1.11	0.24	
**Mother BMI**	0.99	0.95–1.03	0.52		1.04	0.95–1.13	0.42		0.99	0.95–1.03	0.71		1.01	0.97–1.05	0.64		0.97	0.90–1.04	0.33		1.01	0.96–1.06	0.64	
**Father BMI**	1.01	0.98–1.05	0.41		1.00	0.93–1.07	0.99		1.03	1.00–1.07	0.052		0.99	0.96–1.02	0.53		0.96	0.91–1.02	0.20		1.03	0.99–1.07	0.11	
**Mother Education**																								
**Secondary high school or lower**	1.17	0.83–1.66	0.37	0.66	1.41	0.67–2.98	0.37	0.59	1.21	0.85–1.72	0.28	0.36	1.10	0.79–1.53	0.58	0.15	1.88	1.05–3.39	0.04	0.10	0.84	0.55–1.28	0.42	0.43
**High school**	1.07	0.78–1.46	0.69		1.38	0.70–2.72	0.35		1.25	0.92–1.72	0.16		0.84	0.62–1.13	0.24		1.58	0.91–1.02	0.11		1.07	0.74–1.55	0.72	
**Bachelor’s or higher (ref.)**	-	-	-		-	-	-		-	-	-		-	-	-		-	-	-		-	-	-	
**Father Education**																								
**Secondary high school or lower**	0.91	0.63–1.30	0.59	0.84	0.88	0.40–1.93	0.75	0.51	1.07	0.75–1.54	0.70	0.58	0.90	0.64–1.26	0.53	0.23	0.87	0.48–1.58	0.64	0.32	0.82	0.53–1.28	0.38	0.64
**High school**	0.92	0.68–1.25	0.61		1.26	0.66–2.39	0.49		0.92	0.68–1.25	0.58		1.14	0.86–1.53	0.36		1.23	0.74–2.04	0.44		0.96	0.67–1.38	0.83	
**Bachelor’s or higher (ref.)**	-	-	-		-	-	-		-	-	-		-	-	-		-	-	-		-	-	-	
**Mother Smoking (cigarettes/day)**																								
**0 (ref.)**	-	-	-		-	-	-		-	-	-		-	-	-		-	-	-		-	-	-	
**≥1**	1.09	0.30–3.95	0.90		1.58	0.19–13.27	0.68		1.65	0.48–5.68	0.43		1.05	0.31–3.53	0.94		1.09	0.13–8.94	0.94		2.15	0.52–8.90	0.29	
**Father Smoking (cigarettes/day)**																								
**0 (ref.)**	-	-	-	0.12	-	-	-	0.53	-	-	-	0.66	-	-	-	0.04	-	-	-	0.97	-	-	-	0.02
**<5**	1.21	0.94–1.57	0.14		1.26	0.72–2.19	0.42		1.11	0.86–1.44	0.42		0.94	0.74–1.20	0.64		0.98	0.65–1.50	0.94		1.19	0.88–1.60	0.26	
**5–10**	1.36	0.99–1.88	0.06		0.99	0.47–2.07	0.98		1.21	0.87–1.67	0.26		1.33	0.97–1.83	0.07		0.87	0.50–1.53	0.64		0.90	0.60–1.34	0.60	
**>10**	1.38	0.96–1.99	0.08		1.63	0.80–3.31	0.18		1.13	0.78–1.62	0.53		0.73	0.52–1.03	0.07		0.96	0.53–1.75	0.89		0.46	0.26–0.81	0.01	
**Household income (RMB)**																								
**< 80,000 (ref.)**	-	-	-	0.04	-	-	-	0.27	-	-	-	0.10	-	-	-	0.19	-	-	-	0.02	-	-	-	0.83
**80,000–150,000**	1.46	1.09–1.97	0.01		1.44	0.72–2.86	0.30		1.30	0.97–1.74	0.08		1.28	0.97–1.68	0.08		0.53	0.34–0.83	0.01		0.98	0.68–1.40	0.90	
**> 150,000**	1.30	0.94–1.79	0.12		1.80	0.88–3.72	0.11		1.04	0.76–1.43	0.80		1.11	0.83–1.50	0.48		0.65	0.40–1.05	0.08		1.07	0.73–1.58	0.73	
**Breastfeeding (months)**																								
**0**	1.19	0.88–1.60	0.26	0.51	1.49	0.81–2.73	0.20	0.34	0.97	0.72–1.31	0.83	0.63	0.80	0.61–1.05	0.12	0.13	0.95	0.58–1.57	0.85	0.88	0.99	0.69–1.42	0.96	0.30
**<6**	1.08	0.80–1.45	0.61		1.36	0.74–2.49	0.33		0.86	0.63–1.17	0.33		0.80	0.60–1.06	0.12		1.12	0.68–1.84	0.67		1.31	0.92–1.85	0.13	
**≥6 (ref.)**	-	-	-		-	-	-		-	-	-		-	-	-		-	-	-		-	-	-	
**Physical activity (minutes/day)**	1.00	1.00–1.00	0.91		1.01	1.00–1.01	0.01		1.00	0.99–1.00	0.42		1.00	0.99–1.00	0.27		1.00	1.00–1.01	0.76		1.00	1.00–1.01	0.44	

^a^ consumption as reference.

**Table 7:** Association between consumption frequency of fast food, snacks, and beverages and factors from linear regression models.

Variables	Fast food			Snacks			Beverages		
	B	95% CI	*p*-Trend	B	95% CI	*p*-Trend	B	95% CI	*p*-Trend
**Age**	0.01	0.004–0.02	0.00	0.06	0.04–0.08	0.00	0.05	0.03–0.07	0.00
**Gender**	0.01	−0.03–0.04	0.76	0.11	0.02–0.19	0.01	−0.03	−0.11–0.05	0.52
**Mother BMI**	0.00	−0.01–0.002	0.24	−0.02	−0.03–−0.003	0.02	−0.01	−0.03–0.01	0.23
**Father BMI**	0.01	0.001–0.01	0.03	0.00	−0.02–0.01	0.61	0.00	−0.01–0.01	0.99
**Mother education**	−0.01	−0.04–0.01	0.25	−0.03	−0.10–0.03	0.39	−0.13	−0.19–−0.06	0.00
**Father education**	0.00	−0.03–0.03	0.96	0.07	−0.00–0.14	0.06	0.03	−0.04–0.09	0.47
**Mother smoking (cigarettes/day)**	−0.04	−0.23–0.15	0.68	0.17	−0.36–0.71	0.52	0.39	−0.09–0.88	0.11
**Father smoking (cigarettes/day)**	−0.01	−0.02–0.01	0.26	0.08	0.03–0.12	0.00	0.02	−0.02–0.06	0.27
**Household income (RMB)**	−0.02	−0.04–0.01	0.18	0.08	0.02–0.14	0.01	0.05	−0.01–0.11	0.12
**Breastfeeding (months)**	0.02	−0.00–0.04	0.06	0.01	−0.05–0.07	0.74	0.04	−0.01–0.10	0.13
**Physical activity (minutes/day)**	0.00	−0.001–0.001	0.84	0.00	0.001–0.004	0.00	0.00	0.00–0.01	0.00

-: minus value.
